# Early Growth Stage Characterization and the Biochemical Responses for Salinity Stress in Tomato

**DOI:** 10.3390/plants10040712

**Published:** 2021-04-07

**Authors:** Md Sarowar Alam, Mark Tester, Gabriele Fiene, Magdi Ali Ahmed Mousa

**Affiliations:** 1 Department of Arid Land Agriculture, Faculty of Meteorology, Environment and Arid Land Agriculture, King Abdulaziz University, Jeddah 21589, Saudi Arabia; 2 Plant Breeding Division, Bangladesh Agricultural Research Institute (BARI), Gazipur 1701, Bangladesh; 3 Center for Desert Agriculture, King Abdullah University of Science and Technology, Thuwal 23955, Saudi Arabia; mark.tester@kaust.edu.sa (M.T.); Gabriele.Fiene@kaust.edu.sa (G.F.); 4 Department of Vegetables, Faculty of Agriculture, Assiut University, Assiut 71526, Egypt

**Keywords:** seedling traits, PCA, indices, cluster analysis, antioxidants, salt stress

## Abstract

Salinity is one of the most significant environmental stresses for sustainable crop production in major arable lands of the globe. Thus, we conducted experiments with 27 tomato genotypes to screen for salinity tolerance at seedling stage, which were treated with non-salinized (S1) control (18.2 mM NaCl) and salinized (S2) (200 mM NaCl) irrigation water. In all genotypes, the elevated salinity treatment contributed to a major depression in morphological and physiological characteristics; however, a smaller decrease was found in certain tolerant genotypes. Principal component analyses (PCA) and clustering with percentage reduction in growth parameters and different salt tolerance indices classified the tomato accessions into five key clusters. In particular, the tolerant genotypes were assembled into one cluster. The growth and tolerance indices PCA also showed the order of salt-tolerance of the studied genotypes, where Saniora was the most tolerant genotype and P.Guyu was the most susceptible genotype. To investigate the possible biochemical basis for salt stress tolerance, we further characterized six tomato genotypes with varying levels of salinity tolerance. A higher increase in proline content, and antioxidants activities were observed for the salt-tolerant genotypes in comparison to the susceptible genotypes. Salt-tolerant genotypes identified in this work herald a promising source in the tomato improvement program or for grafting as scions with improved salinity tolerance in tomato.

## 1. Introduction

Among the environmental stresses, elevated salinization is identified as a severe detrimental one, mostly in desert and semi-desert climatic countries, triggering large yield losses for most of the domesticated plants across the globe. High salinity influences more than 800 million hectares of global area [1]. More than 20% of the earth’s irrigated land is impaired with salt, with a growing trend as a consequence of climatic change and excess usage of underground water resources. Generally, impaired water uptake, germination disruption, stunted growth, photosynthesis retardation, oxidative stress, and yield reduction are the key effects of salinity on crop plants. Salt stress impacts nearly all component in plants—physiological, biochemical, and molecular mechanisms—and causes a considerable reduction in biomass production and ultimately the yield [2,3,4]. Major adverse effects of salt stress are decreases in hydraulic potential leading to osmotic stress, ionic imbalance due to Na^+^ and Cl^−^ harmfulness on plants’ physiological and biochemical functions, cellular energy dynamics, and disturbance of essential element supply and uptake [5,6,7]. Glycophytic plants, in particular a substantial portion of crop plants, are unable to thrive in soil with 50 mM or above sodium ion (Na^+^) content. Crop yield losses occur when the soil solution reaches around 40–50 mM NaCl with 0.2 MPa osmotic pressure [6,8]. Surplus deposited salt in plants may block the function of enzymes and lead to leaf mortality. Salt can also negatively impact the functions of chloroplasts and leaf metabolites, as well as have a detrimental effect in the functions and units of photosynthesis in plants [6].

Plant reactions to excess salt may be represented by two distinct steps. Firstly, the shoot ion-independent reaction, which is believed to be linked to Na^+^ sensing, with signaling occurring within minutes to days [9]. Here, salt stress affects plant osmotic relationships, culminating stomatal shutdown and the reduced leaf proliferation [10]. Secondly, the ion-dependent reaction, which progresses over a prolonged time from a few days to weeks, and includes effects due to ions accumulating to harmful levels, primarily in older leaves, which leads to early leaf senescence, decreased yield, or even mortality of the plant [6]. Halophytes adapt to stress conditions through diverse cellular processes such as ion compartmentalization, synthesis of osmoprotectants and osmolytes, antioxidant enzyme regulation, and plant hormone synthesis [11,12,13]. Molecular strategies include salt overly sensitive (SOS) transporters for ionic homeostasis, amino acid synthesis, plant hormone networking, protein-encoding gene control and expression, photosynthetic units, and free radical neutralization [8].

Tomato (*Solanum lycopersicum* L.), a member of the Solanaceae family, is amongst the most significant crops, largely cultivated in open fields as well as under protective culture. It is one of the major and popular horticultural crops for its diversified uses and is also rich in nutritional and antioxidental bioactive compounds [14]. Moreover, tomato yield and fruit quality are significantly affected by abiotic stresses and cultural practices [14,15]. Despite widespread adaptability and dissemination, the yield of tomato has been curtailed as a result of raised soil or irrigation water salinity [16]. The majority of tomato cultivars have the genetic potential to tolerate mild to medium salt stress [17]. Every plant growth and development stage (seed germination, vegetative and reproductive growth) demonstrate sensitivity to salinity stress, leading to decreased crop performance and yield [18,19]. However, in many crops, studies have revealed that salinity tolerance at the early growth stages is of more importance than at the later plant stages [19,20]. Maintenance of growth, which is defined as an increase in mass mainly at early growth stage, has been widely known as an important indicator of salinity tolerance [21].

To assess the salinity tolerance of a crop, one should determine the contributing character(s) depending on its predominant response in contrasting salt stress environments [21,22]. From this perspective, studies have been carried out considering physio-morphological attributes, leaf metabolites, and K^+^/Na^+^ and Ca^2+^/Na^+^ ion ratios [23,24], and fresh and dry mass of shoots and roots at vegetative growth period were recommended as evaluation standard for salinity stress reactions [19,21]. Growth and biomass yield, leaf area and leaf number at saline conditions [25,26], and selective uptake of K^+^ over Na^+^ are also suggested as the most significant criteria for the screening of salinity tolerance in tomato [27].

As salinity tolerance is a complicated polygenic crop parameter, it is hard to predict salinity tolerance on the basis of a specific trait [20]. Simple correlation analysis is preferred, with pairwise regression of attributes, but it may not give expected results with complex salinity traits. Hence, the use of principal component analysis (PCA) can illustrate the most significant plant attributes governing salt tolerance [28]. In PCA, a three-way interaction plot aided with clustering can provide efficient prediction of the responses of salinity stress tolerance of the cultivars relying on trait associations existing between morpho-physiological traits and Na^+^ stress [29,30]. To select relatively stress tolerant genotypes, various strategies have been proposed. A common approach is a choice of genotypes on the basis of their biomass or yield performance in stress conditions relative to control or non-stress conditions. Many other studies have suggested a different approach to identifying tolerant genotypes efficiently for abiotic stress on the basis of some stress indices or selection criteria. These stress indices can provide reliable indication to evaluate the genotypic performance of a crop on the basis of the production biomass or yield [20,31,32].

Elevated salt stress has been observed for stimulation of reactive oxygen species (ROS) generation and aggregation within the stressed organs, plant tissues, or cellular spaces [33,34]. These various forms of antioxidants build a complex mechanism for neutralizing or scavenging ROS generated in the plant cells, tissues, or organs [35], as well as to save plant cells or tissues from ROS generated as a result for oxidative injury [36]. The protection from oxidative stress injury by scavenging ROS is an effective solution to harsh environmental conditions for crops [37].

The current research was therefore undertaken to screen 27 tomato genotypes for salt stress tolerance, taking into account the morpho-physiological traits at the vegetative stage. In addition, different biochemical parameters were studied in selected genotypes with varying degrees of salt tolerance for identification of important variables governing salinity tolerance in tomato and utilizing the tolerant genotypes as scions for grafting or in breeding for improved salt tolerance.

## 2. Results

### 2.1. Variability and Correlation Analysis

Analysis of variance suggested that all the genotypes with respect to different morpho-physiological features were substantially different from each other. Important discrepancies between treatment levels for all of the traits were also observed when comparing salinity levels (control and salt stress treatment) (Table S1).

A higher response rate to exposed salt stress was observed in all tomato genotypes with raised saltiness in growing media. The control without any stress was successful in growing more leaf area and higher leaf numbers, highly stable leaf tissue membrane, higher leaf relative water content, longer and heavier shoots, higher relative chlorophyll content or soil plant analysis development (SPAD) values with more chlorophylls (a, b), and high K^+^/Na^+^ ratios compared to the salt stress treated plants. At 200 mM irrigation water salinity, all of these characteristics decreased to a significant amount relative to the non-stressed or normal conditions. With increased salinity stress, root length (RL), fresh and dry root weight (FRW, DRW), root to shoot length and dry mass ratio (RSR, RTSDWR), and Na^+^ accumulation in leaves were raised predominantly. As a result of higher sodium accumulating in leaves, the lower ratio of K^+^/Na^+^ was found in salt-treated plants, but for most of the genotypes, the root to shoot length and weight ratio (RSR or RSDWR) were higher relative to the control with non-salinized conditions (Figure S1).

Visual plant damage score for severity of salt susceptibility was lower in tomato genotypes Saniora, BARI hybrid-8, Mongal Raja, P.Sartaj, and Epoch. However, higher susceptibility for plant damage was observed in genotypes P.Guyu, Swaraksha, and BARI-17. Again, in higher salt stress, percent reduction for total dry matter or biomass production was also lower in Saniora, BARI hybrid-8, Mongal Raja, P.Sartaj, and BARI-11, but higher biomass reduction was observed in P.Guyu, Outta, Swaraksha, and BARI-17 (Figure S2).

**Figure 1:**
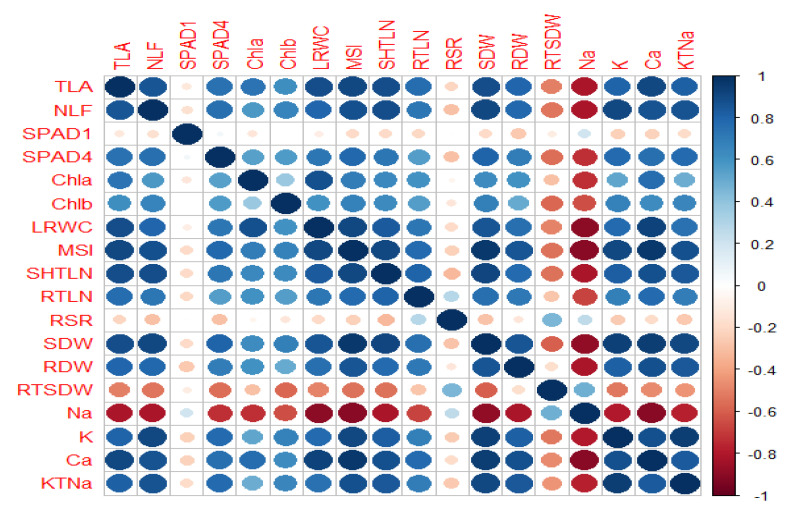
Correlation co-efficient between 18 physio-morphological traits in 27 tomato genotypes at the seedling stage under non-salinized control (S1) with 18.2 mM NaCl and salinized (S2) with 200 mM NaCl conditions during 2018–2019 (TLA = total leaf area per plant (cm^2^), NLF = number of leaves per plant, SPAD1 = soil plant analysis development (SPAD) value at first week of salt stress, SPAD4 = SPAD value at fourth week of salt stress, Chla = chlorophyll a concentration of fresh leaves (mg g^−1^ fresh mass), Chlb = chlorophyll b concentration of fresh leaves (mg g^−1^ fresh mass), LRWC = leaf relative water content (%), MSI = membrane stability index of fresh leaves, SHTLN = shoot length (cm), RTLN = root length (cm), RSLR = root to shoot length ratio, SDW = shoot dry weight per plant (g), RDW = root dry weight per plant (g), RSDW = root to shoot dry weight ratio, Na = sodium content in leaf (mmol/g DW), K = potassium content in leaf (mmol/g DW), Ca = calcium content in leaf (mmol/g DW), KTNa = potassium to sodium content ratio in leaf. Pearson matrix of correlation of the studied attributes under control (S1) and salt stress (S2) (200 mM NaCl) treatments (*n* = 4). Large circles denote strong relationships and smaller circles denote weaker relationships. The color scale indicates the extent of correlation, where 1 denotes completely positive relationships in dark blue and −1 denotes completely negative correlation in dark red between two traits. Only significant correlations are shown (*p* = 0.05).

Correlation coefficient studies (Table S1 and Figure 1) demonstrated significantly positive associations between the total leaf area, number of leaves per plant, SPAD value at fourth week of salt stress, leaf chlorophyll (a, b) concentrations, leaf relative water content (LRWC), membrane integrity index (MSI), shoot and root length and dry weight, leaf potassium ion concentration, leaf calcium concentration, and potassium to sodium ratio of leaf, which indicated that any increment of these traits will enhance the salinity stress tolerance of tomato genotypes. Through studying the relationship of Na^+^ deposition in tomato leaves due to increased salt stress with all other physio-morphological characters, we revealed that Na^+^ had strong negative correlations with almost all studied traits, except RSR and RSDW because of its negative interactions on them. We found that the SPAD value at first week (SPAD1) of salt stress demonstrated non-significant relationships with all other traits, and thus for the PCA analysis, this character was excluded. For all of the morphological as well as the physiological parameters, highly negative effects of leaf Na^+^ concentration were observed. Therefore, these plant characteristics should be given priority for screening of salinity tolerance in tomato.

### 2.2. PCA for Physio-Morphological Traits

**Figure 2:**
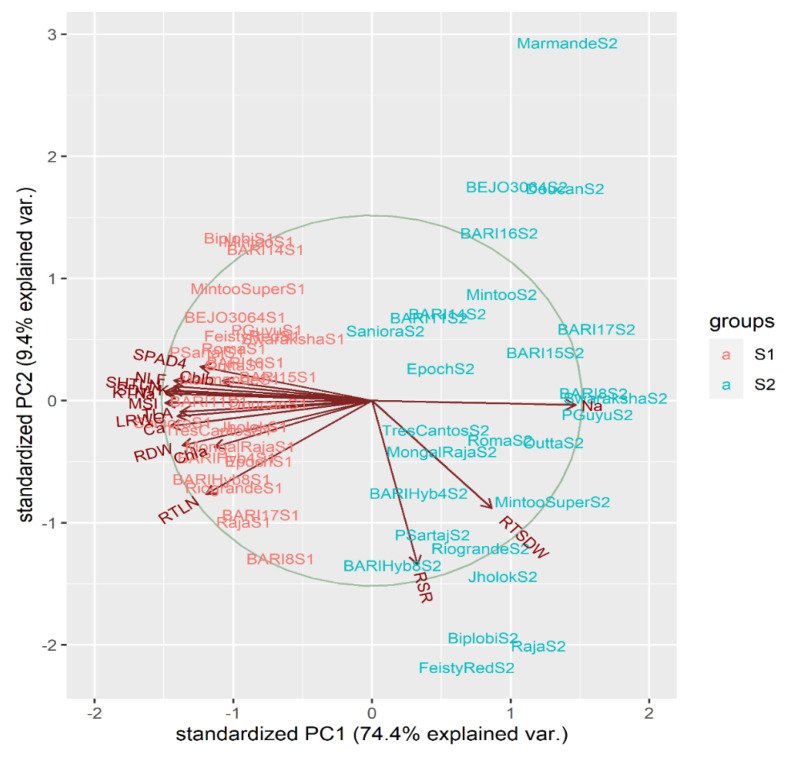
Three-way principal component biplot (PC-biplot) for genotypes × salinity levels × physio-morphological attribute responses with 27 tomato accessions at the early growth stage under non-salinized control (S1) with 18.2 mM NaCl and salinized (S2) with 200 mM NaCl conditions during 2018–2019. The two PCs (PC1 and PC2) described 74.4% and 9.4% of the variance, respectively. Arrows indicate the intensity of the plant characters effects in the first two components. The arrows’ direction and distance show the extent of traits influencing the first two PCs. Aligned vectors suggested a significant positive relationship between the two plant characters. Vectors at perpendicular and opposing forces revealed no association and negative association, respectively (TLA = total leaf area per plant (cm^2^), NLF = number of leaves per plant, SPAD1 = SPAD value at first week of salt stress, SPAD4 = SPAD value at fourth week of salt stress, Chla = chlorophyll a concentration of fresh leaves (mg g^−1^ fresh mass), Chlb = chlorophyll b concentration of fresh leaves (mg g^−1^ fresh mass), LRWC = leaf relative water content (%), MSI = membrane stability index of fresh leaves, SHTLN = shoot length (cm), RTLN = root length (cm), RSLR = root to shoot length ratio, SDW = shoot dry weight per plant (g), RDW = root dry weight per plant (g), RSDW = root to shoot dry weight ratio, Na = sodium content in leaf (mmol/g DW), K = potassium content in leaf (mmol/g DW), Ca = calcium content in leaf (mmol/g DW), and KTNa = potassium to sodium content ratio in leaf).

In PCA, every other salinity level (S1 = control with 18.2 mM and S2 = 200 mM NaCl irrigation water salinity) was simultaneously used to examine the association between morpho-physiological characteristics (generated by genotypic response impact towards salt stress) and among the genotypes (Figure 2). The average values for the 17 seedling attributes were utilized for PCA. After obtaining the eigenvalues for these 17 principal components (PCs), we noticed that the first two PCs (PC1 and PC2) had eigenvalues higher than 1 (Table S3). The first two PCs explained a total variability of 83.8%; however, for the first three PCs as a whole, the total variance output was 88.2%. The data matrix table containing 17 (traits) for 27 (genotypes) was generated and tabulated by R to get all 17 principal components and their loadings for all corresponding characters (Figure 2 and Table S3). Individual PC vectors allocated variance scale values to all characters as a consequence of these traits to the entire variability. These vectors were utilized to illustrate the principal component biplot (PC-biplot) using R.

The PC-biplot of physio-morphological characteristics of tomatoes (Figure 1) reflected two forms of assembling between characteristics; one category comprised almost all plant characteristics—i.e., total leaf area (TLA), number of leaves (NLF), SPAD value at fourth week of salt stress (SPAD-4), chlorophyll a concentration (Chl.a), Chlorophyll b concentration (Chl.b), leaf relative water content (LRWC), membrane stability index (MSI), shoot length (SL) and dry weight (SDW), leaf potassium (K) and calcium (Ca) content, and potassium to sodium content ratio (KTNa), which in leaf were fairly close to one another, and the second category comprised sodium content in leaf (Na), root to shoot length ratio (RSR), and root to shoot dry weight ratio (RTSDW). The opposing vectors of Na and physiological characteristics (TLA, NLF, MSI, LRWC, Chl.a, Chl.b, K, KTNa) contained significant negative associations among themselves. Shoot morphological (SL, FSW, DSW) and leaf physiological trait (MSI, Chl.a, Chl.b, KTNa.R, Ca) vectors were reasonably close to each other, suggesting positive links among themselves. However, the physiological attributes themselves were in a clearly significant positive correlation because of their longitudinal identical vectors. Na vector was also opposite to shoot morphological characteristics (SL, FSW, DSW) but showed positive relationships with RSR and RTSDW.

The PC-biplot distributed the genotypes in order to better comprehend the genetic potentiality of responses to salt stress treatment with all morpho-physiological parameters as well as for sensible comparison of salt stress levels. The red-colored genotypes reflect their responses to all characteristics at the control level, and the salt stress response is shown by the blue-colored genotypes. At the control treatment, genotypes displayed maximum values of morpho-physiological characteristics since they were clustered in contrast to Na, RTSDW, and RSR.

Among genotypic responses to control treatment with 18.2 mM irrigation water salinity, BARI-11, P.Sartaj, Roma, Marmande, Outta, Saniora, Tres Cantos, and BEJO 3064 were observed with maximum character vectors, with the exception of Na, RSLR, and RTSDW vectors. PCA biplot demonstrated that genotypes were fairly near to each other at non-salinized condition and exhibited no noticeable decline of physio-morphological characters values, whereas genotypes shifted towards Na, RSR, and RTSDW vectors with salinized treatment with 200 mM NaCl in irrigation water. At salt stress treatment, Na had checked physiological traits of most of genotypes; however, Biplobi, Jholok, Raja, Feisty Red, BARI hybrid-8, and Rio-grande had better mean values for root- and shoot-associated traits. Some of the tomato varieties, i.e., BARI-8, BARI-15, BARI-17, Swaraksha, P.Guyu, and Outta, at 200 mM salt stress treatment showed maximum distance vectors for Na and RSDWR. The affection of these genotypes with Na vector strongly suggests higher Na+ amassing in their leaves, which distressed the plant physiological activities. These tomato varieties are extremely salt-sensitive relative to Saniora, BARIhybrid-8, P.Sartaj, BARI-11, BARI hybrid-4, and Epoch, which were remarkably salinity stress-tolerant owing to their exceptional characteristics responses at 200 mM NaCl saline irrigation.

### 2.3. PCA for the Salt Tolerance Indices

**Figure 3:**
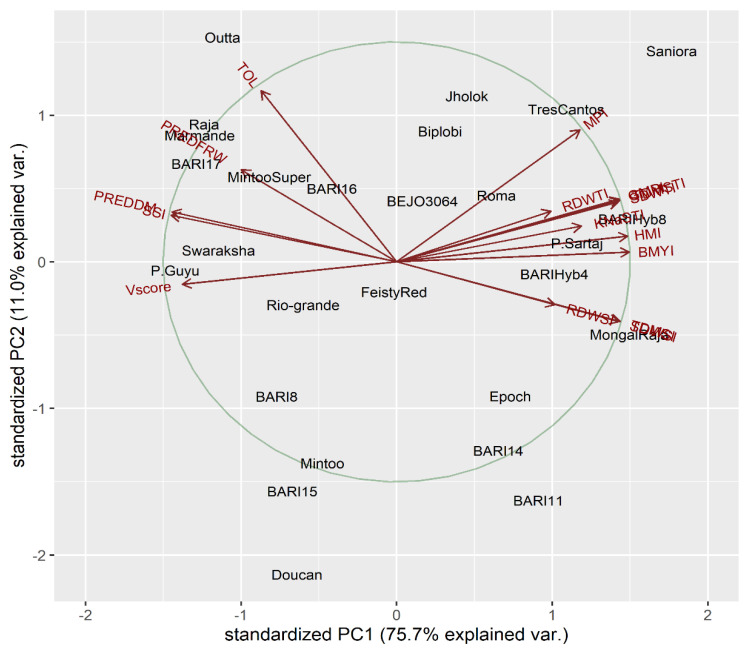
Principal component biplot for genotypes × salt tolerance index traits for 27 tomato genotypes at the early growth stage with non-salinized control (S1) with 18.2 mM NaCl and salinized (S2) with 200 mM NaCl conditions during 2018–2019 (VScore = visual salt damage score, TOL = tolerance index, SSI = stress susceptibility index, PREDFRW = percent reduction of fresh weight, PREDDM = percent reduction of dry weight, MPI = mean productivity index, HMI = harmonic mean index, GMPI = geometric mean productivity index, SDWSTI = shoot dry weight stress tolerance index, RDWSTI = root dry weight stress tolerance index, TDMSTI = total dry matter stress tolerance index, KNaSTI = potassium to sodium ion ration tolerance index, SDWSI = shoot dry weight salt tolerance index, RDWSI = root dry weight salt tolerance index, TDMSI = total dry matter salt tolerance index, and BMYI = biomass yield index).

The means of treatments for all salt tolerance index-related traits were in strong relationships and were used for PCA (Figure S3). The data matrix table comprising 16 (tolerance index traits) × 27 (genotypes) was produced and outlined by R to extract all 16 PCs and their loadings for each corresponding variable (Table S5). The PC-biplot of tomato salt tolerance index-related parameters (Figure 3) illustrates two distinct forms of aggregation among characters—one group containing almost all salt tolerance index traits, i.e., visual leaf damage score (Vscore), stress tolerance (TOL), stress susceptibility index (SSI), percent reduction of fresh weight (PREDFRW), and percent reduction of dry weight (PREDDM), which were very close and overlapping each other, and a second group containing mean productivity index (MPI), harmonic mean productivity index (HMI), geometric mean productivity index (GMPI), stress tolerance indices (STI) for SDW (SDWSTI), STI for RDW (RDWSTI), STI for TDM (TDMSTI), STI for KTNa (KNaSTI), salt tolerance index (SI) for SDW (SDWSI), SI for RDW (RDWSI), SI for TDM (TDMSI), and biomass yield index (BMYI).

Both the groups exhibited a strong negative correlation between themselves but among the groups the traits showed strong positive linkage. After obtaining the eigenvalues for all 16 PCs, we noticed that the first two PCs had eigenvalues higher than 1 (Table S5). Percent variances and cumulative variances for PCs were gained depending on the corresponding eigenvalues of each PC (Table S5). In PCA-biplot, PC1 described about 75.7%, and PC2 represented 11.0%, with both PCs exhibiting 86.7% of variance. Moreover, with the first three PCs, the cumulative contribution of variability was 92.0% (Figure 3). The PCA chronologically ordered the tolerant and susceptible tomato genotypes on the basis of their respective tolerance indices. The PC1 reflected nearly two-thirds of the variance, and thus we were able to conceptualize all genotypes relying on PC1 only. In the PCA individual graph, the genotypes residing more on the right edge were found to be quite salt-tolerant and the genotypes positioned at more left side were identified as more susceptible to salt stress (Figure S4).

Considering the PC1, the order of relative salinity tolerance of the tomato genotypes was found as Saniora > BARI hybrid-8 > Mongal Raja > P.Sartaj > Tres Cantos > BARI hybrid-4 > BARI-11 > Epoch > Roma > BARI-14 > Jholok > Biplobi > BEJO 3064 (Figure 3 and Figure S2). However, on the contrary, the relative ranking of salt-susceptibility of the studied genotypes were as follows: P.Guyu > BARI-17 > Marmande> Raja > Swaraksha > Outta > MintooSuper > BARI-8 > Doucan > BARI-15 > Rio-grande > Mintoo > BARI-16 > Feisty Red (Figure 3 and Figure S4 and Table S6). Thus, for ranking the overall salinity tolerance of the studied genotypes, the highest PCA individual coordinate score (PC1) was for Saniora and the lowest score was for P.Guyu (Table S6).

### 2.4. Hierarchical Cluster Analysis for Salt Tolerance Indices

**Figure 4:**
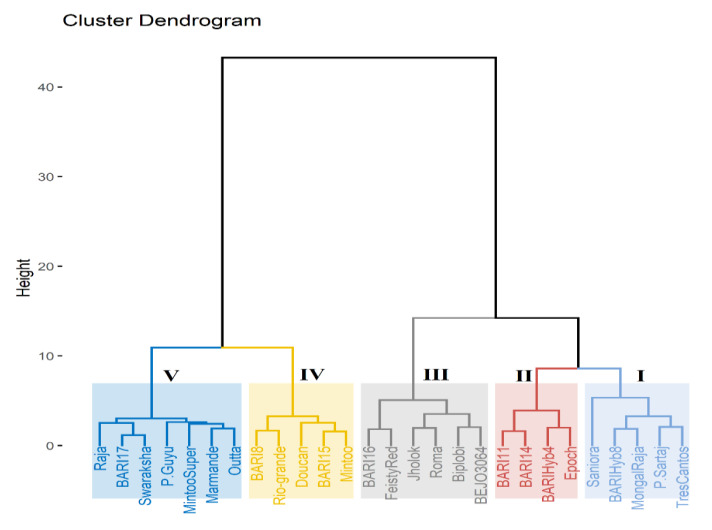
Dendrograph derived by Ward’s methods of cluster analysis for salt tolerance indices with 27 tomato genotypes at the vegetative stage under non-salinized control (S1) with 18.2 mM NaCl and salinized (S2) with 200 mM NaCl conditions during 2018–2019.

The Euclidean distance-based hierarchical clustering organized the tomato genotypes into five key subclusters: clusters I, II, III, IV, and V (Figure 4). Cluster I contains genotypes with the lowest decrease in growth parameters and with higher mean salinity tolerance score under salinity environment, and thus these genotypes could be classified as highly salt-tolerant. Therefore, the genotypes Saniora, BARI hybrid-8, Mongal Raja, and Tres-cantos included in this cluster were found to be fairly salt-tolerant. There were four genotypes, notably, Epoch, BARI hybrid-4, BARI-11, and BARI-14, in cluster II, which demonstrated a lower decrease in growth variables in salt stress, and thus this cluster was categorized as a moderate salt-tolerant cluster. The genotypes grouped in cluster III showed medium decrease of contributing traits with medium salt tolerance score, and therefore these were identified also as medium-to-lower salinity tolerant genotypes. The genotypes aggregated in cluster IV and in cluster V had a marked decrease in growth performance under salt stress, and these two clusters were therefore classified as extremely or moderately salt-susceptible clusters. Thus, the genotypes accommodated in cluster V—Outta, Marmande, Mintoo Super, P.Guyu, Swaraksha, Raja, and BARI-17—were identified to be susceptible or highly susceptible to salinity stress.

### 2.5. Salt Tolerance and Characterization of Biochemical Traits

**Figure 5:**
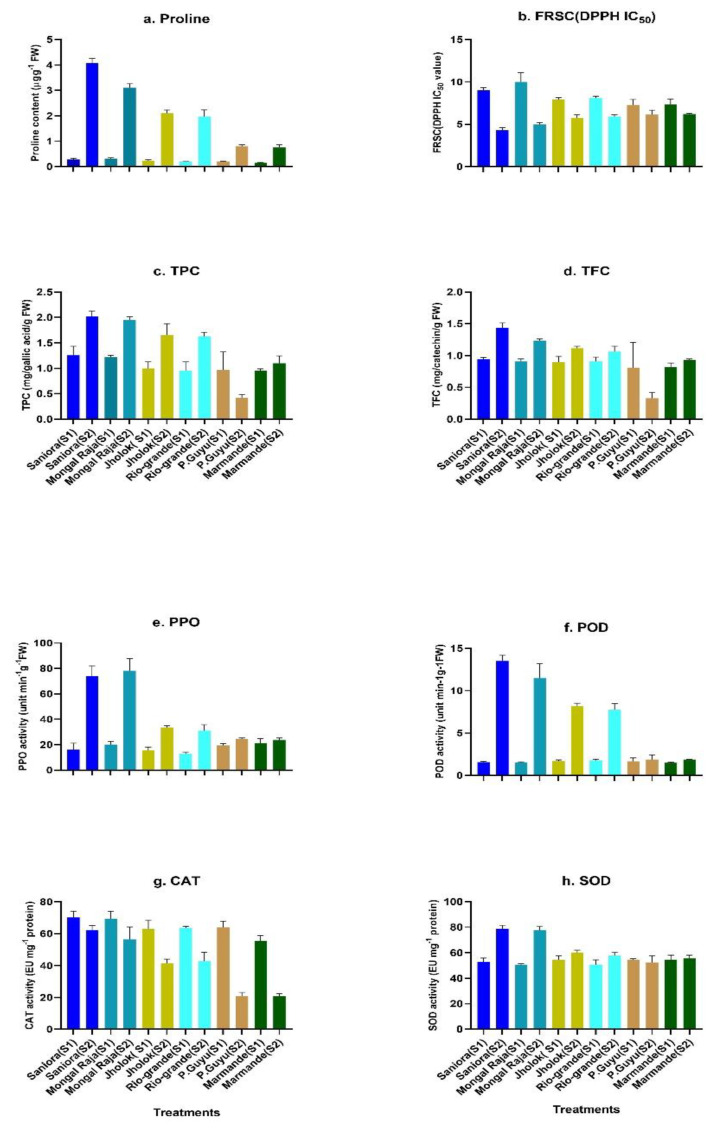
Biochemical parameters—(**a**) proline content, (**b**) free radical scavenging capacity (FRSC) in 1,1-diphenyl-2-picrylhydrazyl (DPPH) IC_50_, (**c**) total phenol content (TPC), (**d**) total flavonoid content (TFC), (**e**) polyphenol oxidase (PPO) enzyme activity, (**f**) peroxidase (POD) enzyme activity, (**g**) catalase (CAT) enzyme activity, and (**h**) superoxide dismutase (SOD) enzyme activities of three tomato genotypes with varying tolerance levels exposed to stress treatments with S1 = control (18.2 mM NaCl) and S2 = salt (200 mM NaCl) (mean ± SD, *n* = 3 with three replicates; bars with similar letters are statistically identical with *p* ≤ 0.05).

The measurements of biochemical determinants of salinity-stressed leaves of six tomato genotypes with varying levels for salinity tolerance were exhibited in Figure 5. The higher value for proline accumulation was noticed in tomato genotypes Saniora, Mongal Raja, and Jholok, but the salt-sensitive P.Guyu and Marmande showed the lowest value for proline in increased salinity stress treatment. Again, for the free radical scavenging capacity (FRSC), the 1,1-diphenyl-2-picrylhydrazyl (DPPH) activity in IC_50_ value was maximum for P.Guyu and Marmande, followed by Rio-grande and Jholok. However, the lowest IC_50_ values were observed in Saniora and Mongal Raja under salt stress treatment. Nevertheless, here, the values for P.Guyu, Marmande, Rio-grande, and Jholok were statistically similar, and FRSC was lower for all the six genotypes in salt treatments in comparison with control treatments. The findings of the biochemical responses denoted that under stress tomato genotypes, Saniora and Mongal Raja were recorded as the higher values for total phenol content activity followed by medium salt-tolerant genotype Jholok and Rio-grande in relation to the lowest value in salinity-sensitive genotype P.Guyu and Marmande. Considering total flavonoid content activity, the highest activity was observed in salinity-tolerant tomato genotype Saniora followed by Jholok, and both were statistically the same, but the lowest value was for P.Guyu. In salt stress treatment, the levels of polyphenol oxidase (PPO) and peroxidase (POD) activities were also higher in salinity-tolerant tomato Saniora and Mongal Raja in comparison to salinity-sensitive tomato P.Guyu (Figure 5). Among the genotypes, the higher values for catalase (CAT) activity were noticed in Mongal Raja and Saniora, followed by Rio-grande and Jholok, whereas susceptible P.Guyu and Marmande showed the lower value for CAT activities in salt treatment but in all the cases the values were lower than the control. At the same time, the highest reading for POD activity was also found in Saniora, likewise in Mongal Raja. Again, Saniora demonstrated the highest score for SOD activity, followed by Mongal Raja, and lowest value was for sensitive P.Guyu under salinity treatment.

## 3. Discussion

In a range of crops, salinity tolerance at early growth stages often reflects greater tolerance than at later growth stages. For different crops, including tomatoes [28,29,38], barley [39], wheat [40], maize [41], cotton [42], and rice [43], research has shown that genotypes with enhanced tolerance mechanisms have been selected for their resilience to saline environments at the seedling stage. The aim of our present study was to identify salinity tolerance by phenotyping tomato seedlings using quantitative traits such as reduction in mass of roots and shoots, leaf injury due to salt stress, and tolerance indices derived from dry weights and leaf inorganic ions (K^+^, Na^+^, and Ca^2+^) in order to systematically screen salt-tolerant varieties as well as to understand the biochemical mechanisms of salinity stress tolerance in tomatoes.

The principal component analysis (PCA) was carried out to obtain more precise conclusions for classifying the genotypes with desirable yield characters for crop improvement. To observe the interrelation among variables, we utilized the PCA technique [44,45]. For the raised salinity level, the PCA was best documented to distinguish the salt tolerance potentials of genotypes [28,29]. PC-biplot allowed us to select the best genotype through the genotype–environment response interactions. The strengths of PC-biplot allow it to be a much more powerful tool over other statistical techniques in terms of the genotypic evaluation under varying salinity stress [46]. In our current research, major phenotypic variability was observed with tomato germplasms regarding salt tolerance at the vegetative growth period. Data on 18 morpho-physiological characters were used to draw PC-biplot of 27 tomato genotypes against two levels of salinity. The tolerant tomato genotypes were marked as having a larger OP vector with stress tolerance vectors when a perpendicular was generated from the genotypic point.

Tomato plants have been reported previously as moderately tolerant to salinity stress [47], and variable reactions have been observed in genotypes in stress conditions [28,29,48]. Similarly, in our current study, all morpho-physiological characteristics were depressed with increased irrigation salinity with 200 mM NaCl, except for an increment in Na+ accumulation and root to shoot length and dry mass ratios (RSR and RTSDWR, respectively) (Figure 1). In other studies with enhanced salinity levels, shoot and root lengths, fresh weights, and dry weights declined [28]. In our study, a few of the genotypes were identified with less root damage with increased fresh and dry root mass in comparison to shoot characteristics, which is supported by other studies [49]. Adaptation of tomato plants to salt stress conditions enhance the formation of stress roots or feeder roots to uptake adequate nutrients with water [50], resulting in increment of root development. In contrast to shoot morphological responses at high salt conditions, lower affected root activities contributed to a greater root to shoot length and dry weight ratio (RSR and RTSDWR) at all growth stages of the tomato genotype [29]. A technique of salinity tolerance utilized by halophytic plants to prevent detrimental effects [51] is therefore responsible for a decline in shoot development but enhancement of the root growth. In our study, the similar procedure for NaCl stress tolerance was noticed in certain tomato genotypes such as Biplobi, Jholok, Rio-grande, and Feisty Red.

In enhanced salt stress, the physiological parameters in tomatoes such as chlorophyll-a (Chl.a), chlorophyll-b (Chl.b), SPAD index, leaf relative water content, membrane stability index (MSI), leaf K^+^/Na^+^ ratio (KTNa), and leaf K^+^ and Ca^2+^ concentration were restricted due to contrasting Na vector and all physiological characteristics (Figure 1). Closer physiological trait vectors noticed their strong positive correlation among them. It is reported that salinity stress resulted in osmotic stress that blocks the major cellular activities [5], and plants tend to activate hormonal regulations to generate osmoregulators or osmoprotectors. In a favorable or low-stress environment, osmotic adjustment is operated by K^+^, but ceased K^+^ supply during raised salt stress can result in higher generation of stress hormones [52], which could result in declined stomatal conductance and leaf photosynthetic enzyme activity [53]. The extent of K^+^ outflow showed negative association with MSI [54] and resulting membrane injury, owing to a greater influx of Na^+^ ions. Ca^2+^ is also involved in the protection of cell membrane consistency [55], and salt stress reduces Ca^2+^ activity and induces membrane disruption. Due to stress, chlorophyll function and structure are severely affected, causing membrane damage with restricted photosynthesis [2]. In our present study, SPAD values at the fourth week (SPAD4) clearly showed lower values for most of the tomato genotypes but higher values for tolerant genotypes. Salinity and osmotic stress existed simultaneously [56], exacerbating leaf degradation and decreasing chlorophyll formation [57], which eventually reduced the photosynthetic potential due to the straight impairment between chlorophyll and photosynthesis. Salinity induces decline in photosynthetic pigments, primarily chlorophyll (a, b) [58], which were also observed in the PCA biplot as Na and chlorophyll (Chl.a and Chl.b) vectors demonstrating a significant negative association among themselves. Genotypes with more chlorophyll (a, b) content at increased salinity stress may have greater genetic yield potential, as a study has suggested a positive relationship between yield and chlorophyll containment [59].

The membrane stability index (MSI) was a method for studying drought tolerance [60], but in our study we found a strong negative correlation between MSI and the amassing of leaf Na^+^. This observation was also confirmed by other studies [29]. Genotypes Saniora, BARI hybrid-8, P.Sartaj, BARI-11, BARI hybrid-4, and Epoch have sustained themselves even at high salinity with almost normal physiological trait responses (Figure 2), which might be due to active Na^+^ efflux causing newer photosynthetically active leaves without any negative impact by abscising their older leaves with a greater amount of Na^+^ [61]. Again, a few other tomato genotypes such as Biplobi, Jholok, Raja, Feisty Red, BARI hybrid-8, and Rio-grande have increased root activity that permits them to uptake the required nutrients from the soil under a saline environment.

Thus, for these tomato genotypes, one or both of the major NaCl tolerance mechanisms may render salt stress tolerance at increased salinity. In contrast, susceptible genotypes such as BARI-8, BARI-15, BARI-17, Swaraksha, P.Guyu, and Outta were unable to do so due to high Na+ accumulation, which negatively affects the normal biological processes in tomato plants ending with altered morphology and reduced growth. Such observations have been verified by [19,28,30], where tomato salinity screening was conducted under raised salt stress. Similar morpho-physiological findings were also observed in rice [62], in barley [63], in faba bean [10], and in alfalfa [64].

PCA and hierarchical cluster analysis for reduction percentage of different physio-morphological variables, visual leaf damage scores for salt stress, and salt tolerance indices clearly and precisely identified and grouped tomato genotypes for salt tolerance in five clusters. In the PCA biplot, most tolerant genotypes resided at the right, and susceptible genotypes were placed in left side on the basis of their tolerance and susceptibility levels. Thus, in our present study, PCA biplot separated Saniora, BARI hybrid-8, Mongal Raja, P.Sartaj, and Tres Cantos as salt-tolerant and P.Guyu, BARI tomato-17, Marmande, Swaraksha, Rio-grande, and Outta as salt-susceptible genotypes. At the same time, hierarchical cluster analysis also grouped highly tolerant genotypes (Saniora, BARI hybrid-8, Mongal Raja, P.Sartaj, and Tres Cantos) in cluster I and highly sensitive genotypes (P.Guyu, Marmande, Mintoo Super, and Outta) in cluster V. Salt-tolerant tomato genotypes were identified in PCA analysis with different tolerance indices [30]. Similar observations for screening salt-tolerant genotypes in rice in PCA and cluster analysis were also demonstrated by [62]. Stress tolerance indices also successfully utilized in other crops such as tomato [19,30], wheat [65], barley [63], and alfalfa [64].

The significance of antioxidants by detoxifying reactive oxygen species (ROS) such as superoxide and H_2_O_2_ as an essential aspect of the plant salt stress reaction system has already been documented [66]. The aggregation of ions or osmolytes in the cytosol leads to an essential contribution in both osmoprotection and osmotic adjustment under abiotic stress [6]. In our present study, relatively more salt-tolerant tomato genotypes Saniora and Mongal Raja, medium salt-tolerant genotypes Jholok and Rio-grande, and highly susceptible genotypes Marmande and P.Guyu were used to unveil the biochemical mechanisms of salt tolerance in tomato. For proline accumulation, Saniora had the highest value, and P.Guyu showed the lowest reading. Indeed, our observations showed that greater proline accumulation within the leaves of the tolerant genotypes dramatically increased. Proline is selectively retained in the leaves to safeguard photosynthesis under salinity stress to retain the abundance of chlorophyll and cell turgidity [67]. Proline plays a certain role in ROS materials scavenging [68]. In a study of salinity tolerance screening, Gharsallah et al. [48] also observed the same results of higher proline accumulation in tomato. In a rice salinity tolerance experiment, similar biochemical role of proline was confirmed by Tahjib et al. [62].

Phenolics also play a significant role in plants, functioning as internal parts of cell walls, as well as for the modulation of development and growth functions and securing protection for biotic and abiotic stress [69]. The largest and most complicated subgroup of phenolic compounds is the flavonoid subgroup, with a greater array of cellular activities, mainly the inhibition of cellular lipid peroxidation [70]. In our present study, value for total phenol and flavonoid content was also highest in Saniora and lowest in P.Guyu, which also denoted the underlying role of phenols and flavonoids for enhancing salt stress tolerance in tomato. Again, the value for free radical scavenging capacity (FRSC), i.e., DPPH IC_50_, was lower in tolerant Saniora, followed by Jholok, and higher in susceptible P.Guyu. Normally, higher DPPH IC_50_ values imply lower antioxidant capacity and lower total phenolics content. For our result with these three tomato genotypes with varying salt tolerance level, we can conclude that the particular phenolic compound was not reactive in the present reaction. Similar biochemical responses were also observed in potato [71].

In the increased salinity or other abiotic constraints, CAT is active in the reduction and neutralization of excess H_2_O_2_ [72], and is known in tomato fruits as a major enzyme detoxifying H_2_O_2_ [73]. In our present investigation, activities for CAT with the three tomato genotypes showed significant differences, highest in tolerant genotype Saniora, followed by Jholok, and the lowest in susceptible P.Guyu. Similar findings were also confirmed by [48,52,62].

Similarly, values for POD and SOD activities were also highest in Saniora and lowest in P.Guyu, but for these three tomato genotypes, the values were non-significant, which also indicated lower activities of these two antioxidant enzymes in tomato leaves. Salt stress increased significantly POD and SOD activities in salt-tolerant tomato genotype, which were also validated in several experiments [74,75,76]. These findings were implemented effectively to identify salinity-tolerant genotypes following the same studies [77,78].

## 4. Materials and Methods

### 4.1. Plant Materials and Growing Conditions

The research was carried out during the winter of 2018–2019 with 27 tomato varieties and lines, namely, (1) BARI-8, (2) BARI-11, (3) BARI-14, (4) BARI-15, (5) BARI-16, (6) BARI-17, (7) BARI hybrid-4, (8) BARI hybrid-8, (9) Epoch, (10) Biplobi, (11) Mongal Raja, (12) Mintoo, (13) Mintoo super, (14) Jholok, (15) Swaraksha, (16) Rio-grande, (17) Raja, (18) P.Sartaj, (19) P.Guyu, (20) Roma, (21) Tres Cantos, (22) Marmande, (23) Feisty Red, (24) BEJO 3064, (25) Outta, (26) Doucan, and (27) Saniora, which were collected from different parts of the globe.

Seeds of all genotypes were sterilized for 10 minutes with a solution of 2.7% sodium hypochlorite, then washed 4 times thoroughly with double-distilled water. Plastic germi-nation trays containing 36 blocks of peat moss and perlite growing media (2:1) were used to plant tomato seeds. There were 4 seeds in each of the blocks. Tomato seedlings (at the 27th day of sowing) were separately moved to plastic pots of 25 cm in diameter, having a peat and perlite ratio of 3:1 with prevailing outside temperature ranging from 20 to 29 °C and relative humidity (RH) of 53–61% (day/night).

### 4.2. Experimental Design, Salt Stress Application, and Nutrient Management

This study was carried out with a completely randomized block design (RBD) with 4 replications, wherein each replication had 3 tomato seedlings. Two levels of salinity control (S1) with fresh tap water (18.2 mM NaCl) and salt stress (S2) (200 mM NaCl) were applied with irrigation water for proper assessment of each physio-morphological character responses of the studied tomato genotypes. For stress application, 50 mM NaCl with irrigation water was applied at the first day on seedlings aged 31 days and increased subsequently with more 50 mM NaCl at every alternative day, and on the seventh day with maximum 200 mM saline irrigation. This highest stress was continued for 3 weeks, and in between this time, excess nutrient solution with fresh water irrigation was applied in the pots to prevent accumulation of excess NaCl to the growing media. The electrical conductivity (EC) and pH were monitored after collecting the leached solution from the pots to ensure similar quality with the applied treatments. To maintain Ca^2+^ activity, a lower concentration of CaCl_2_ was supplemented with salt stress medium as per the suggestion of [24,49]. Nutrients were applied with irrigation water to all control and salt-treated plants, namely, total nitrogen (N) (20%), phosphorous pentoxide (P_2_O_5_) (20%), potassium oxide (K_2_O) (20%), iron (Fe) EDTA (0.060%), manganese (Mn) EDTA (0.050%), zinc (Zn) EDTA (0.012%), copper (Cu) EDTA (0.003%), boron (B) water soluble (0.020%), and molybdenum (Mo) water soluble (0.003%) (Atlantic Agricola, Spain). Plant protection measures were also taken carefully. Salinity stress was continued up to the 59 days of seedling age, and then the seedlings were raised with normal fresh water irrigation for a week and collected for further analysis as per the procedure stated by Saade et al. [22], Dasgan et al. [23], and Murillo-Amador et al. [53].

### 4.3. Measurements

#### 4.3.1. Growth Parameters

After measuring the physiological traits, we collected the seedlings and divided them into roots, stems, and leaves. The shoot and root fresh weights were recorded, and afterwards they were dried for at least 3 days at 70 °C. Shoot (SDW), root (RDW), and total (TDW) dry mass weight were taken by utilizing an electric equalization (TE 2148 Sartorius). Soil plant analysis development (SPAD) estimations were taken utilizing SPAD meter (Konica Minolta 502, Tokyo, Japan) from 9:00 a.m. to 11:00 a.m. in fully extended third to fifth leaf. Three plants for every replication, 12 seedling related variables in total, were carefully estimated for every cultivar and treatment. Plant leaf area was recorded following [79]. Visual leaf damage score (Vscore) was recorded with the observations of expert horticulturists following the indicators classified by Dasgan et al. and Gharsallah et al. [23,48].

#### 4.3.2. Relative Leaf Water Content (RLWC)

The RLWC was determined as depicted by Yamasaki and Dillenburg [80]. RWC was resolved on completely extended leaves of a comparative age. The new leaves were gauged and set in distilled water in obscurity for 1 day to rehydrate. Leaf turgid weight was taken. At that point, leaves were oven dried at 80 °C for 2 days, and the dry weight (DW) was recorded. RWC was determined by the accompanying formula:

RLWC (%) = [(Leaf fresh mass − Leaf dry mass)/(Leaf turgid mass − Leaf dry mass)] × 100 based.

#### 4.3.3. Estimation of Percent Yield Reduction

Percent reduction (PR%) was measured following the equation as proposed by El Goumi et al. [81]: PR% = 100 × [1 − (Wsalt/Wcontrol)], where, Wsalt = weight of sample in stressed conditions, and Wcontrol = weight of sample in control or normal conditions.

#### 4.3.4. Membrane Stability Index (MSI)

MSI was estimated by taking completely extended new leaves with 3 plants from every treatment. Samples were washed by utilizing distilled water and dried with tissue paper independently. At that point, 2 g of leaf sample of every treatment per replication was set in a test tube containing 10 mL of distilled water. These were put into a hot water bath for 30 min in 40 °C. Electrical conductivity (EC) of water extract inside the cylinders was recorded with portable EC/TDS (total dissolved solids) meter. EC1 and EC2 values were estimated subsequently by putting the cylinders in a water shower at 40 °C and 100 °C, respectively, and cooling them for 30 min. Both the EC values were utilized to measure leaf MSI for studied tomato genotypes at both stress levels, as described by Sairam et al. [82].

#### 4.3.5. Measurement of Stress Tolerance Indices

On the basis of the total dry matter production or biomass yield of tomato varieties or lines, we calculated the stress tolerance or sensitivity indices. These indices comprised the following: stress susceptibility index (SSI) [83], stress tolerance (TOL) [84], geometric mean productivity (GMP) [85], stress tolerance index (STI) [85], salt tolerance index (SI) [5], mean productivity (MP) [86], and harmonic mean (HM) [87]. The stress tolerance indices were calculated by the following formulae:

Stress susceptibility index, SSI = (1 − (S/C))/(1 − (xS/xC))

Stress tolerance, TOL = C − S

Mean productivity index, MPI = (C + S)/2

Geometric mean productivity index (GMPI) = √ C × S

Harmonic mean productivity index (HMPI) = 2(C × S)/(C + S)

Stress tolerance index, STI = (C × S)/(xC)^2^

Salt tolerance index, SI = S/C

Yield index, YI = S/xS

where S and C denote the dry matter yield of all genotypes at salt-stressed and non-stressed or control treatments, respectively, and xS and xC denote the mean dry matter yield over all genotypes under stressed and non-stressed or control treatments, respectively. STI and SI were calculated for all genotypes for root, shoot, as well as total dry mass, and STI for K^+^/Na^+^ (KNaSTI) as per measurement procedures of Turhan and Seniz [24].

#### 4.3.6. Photosynthetic Pigment Measurement

The extraction of chlorophyll (a, b) was performed following Arnon’s methodology [88], while concentration was calculated by McKinney formulas [89]. The chlorophyll content was measured following the procedure followed by Saida et al. [90], and absorbances were recorded spectrophotometrically at 645 and 663 nm wavelengths for chlorophylls a and b, respectively.

#### 4.3.7. Mineral Analysis

The leaf samples were oven-dried at 70 °C for 72 h and ground with a grinder. Dried and ground sample (50 mg) was taken in a conical flask, and acid mixture HNO_3_ to HClO_4_ mL volume ratio (7:3) was added. Then, the mixture was heated for 1–2 h in a sand bath heater. A total of 50 mL of distilled water was pumped after the mixture was cooled down and then it was filtered with Whatman filter paper. Then, samples were taken in sample tubes. From these samples, the Na^+^, K^+^, and Ca^2+^ analyses were performed using standard solutions by Flame Photometer (Biobase BK-FP6450) and measured in mmol/g dry weight (DW) [23,48].

#### 4.3.8. Determination of Leaf Proline Content

Fresh sample (0.25 g) was mixed in 3% sulfosalicylic acid and centrifugation was performed at 11,500 rpm. The filtrate was placed into test tubes with acid ninhydrin, glacial acetic acid, and phosphoric acid, and after that incubated at 100 °C for 1 h. After cooling down in an ice bath, test tubes were vigorously shaken after adding toluene. After a while, toluene with chromophore reading was recorded by a UV–VIS spectrophotometer at 520 nm [91].

#### 4.3.9. Measurement of Total Phenols and Flavonoids

Total phenol (TP) content was measured in accordance with the methods mentioned by Velioglu et al. [92]. Total flavonoid (TF) content was calculated by the process of Zhishen et al. [93] and reported as mg catechin g^−1^ fresh weight (FW).

#### 4.3.10. Free Radical Scavenging Capacity (FRSC) Assessment

FRSC of methanol extracted fresh tomato leaf samples was measured by utilizing the 1,1-diphenyl-2-picrylhydrazyl (DPPH) methodology by Ao et al. [94]. The absorbance was estimated spectrophotometrically at 517 nm. The inhibition concentration (IC_50_) was described as µg phenolic compound of the representing material that scavenges 50% of initial free radicals. The IC_50_ was estimated with the help of the dose–response graphs.

#### 4.3.11. Enzyme Measurements

Soluble proteins were extracted by following the methodology of Antoniou et al. [95]. The protein content was measured by a procedure outlined by Bradford [96] and by taking bovine serum albumin (BSA) as a standard for the measurement of protein.

Polyphenol oxidase (EC 1.14.18.1) (PPO) enzyme activity was measured by catechol oxidation as proposed by Zhang et al. [97] with slight changes. The catechol oxidation rate was observed at 410 nm and reported as unit min^−1^g^−1^FW.

Peroxidase (EC 1.11.1.7) (POD) activity was calculated on the justification of H_2_O_2_ peroxidation with guaiacol [98]. The rise in absorbance as a result of guaiacol oxidation was recorded at 470 nm and reported as unit min^−1^g^−1^FW.

SOD (EC1.15.1.1) activity was measured by the procedure of Hasanuzzaman et al. [99]. The absorbance was read at 560 nm and described as EU mg^−1^ protein.

CAT (EC1.11.1.6) activity was assessed following the method described by Aebi [100]. The absorbance was recorded at 240 nm, and the CAT activity was reported as EU mg^−1^ protein.

### 4.4. Statistical Analysis

Recorded data for studied traits were assigned to analysis of variance as per procedure described by Steel and Torrie [74] to figure out significant variations among the genotypes with their responses with salinity stress using Statistix 8.1 software [101]. The plant characters that manifested significant genotype versus salinity interaction were again utilized for correlation and principal component analysis (PCA). PCA and clustering were devised using the software R, version 4.0.2 [102]. Graphical presentation of figures of biochemical activities was performed using GraphPad Prism 8.0 [103].

## 5. Conclusions

The findings of our present study showed that salt stress caused substantial reductions in physio-morphological activities of tomato at the seedling stage, but for some genotypes, namely, Saniora, BARI hybrid-8, Mongal Raja, P.Sartaj, and Tres Cantos, the growth attributes were less affected by salt-related toxicities and these genotypes were therefore characterized as highly salt-tolerant. The PCA and cluster analyses also confirmed this outcome, which clearly separated all relatively more salt-tolerant genotypes and grouped them within the same cluster depending on their degree of salt stress tolerance. Furthermore, our findings indicated that the biochemical features of proline content and antioxidants related to salinity tolerance can be perceived as significant indicators for the identification of salt-tolerant tomato genotypes. In further studies, the genotypes identified as highly salinity-tolerant could be used for tomato improvement or by using them as scions or rootstocks for grafting.

## Data Availability

The data presented in this study are available on request from the corresponding authors.

## Supplementary Materials

The following are available online at https://www.mdpi.com/article/10.3390/plants10040712/s1: Table S1: Analysis of variance (ANOVA) (mean square) for physio-morphological traits. Table S2: Correlation co-efficient between 18 physio-morphological traits. Table S3: Principal component (PC) loadings of physio-morphological traits. Table S4: Analysis of variance (ANOVA) (mean square) for salt tolerance index-related traits and percent reduction in total dry weight. Table S5: Principal component (PC) loadings for salt tolerance index-related traits. Table S6: Individual coordinate PC scores for salt tolerance index-related traits and percent reduction in total dry weight. Figure S1: Effect of salinity on 18 physio-morphological traits. Figure S2: Visual salt damage score for severity of salt susceptibility by on a 1–5 scale. Figure S3: Correlation co-efficient between salt tolerance index-related traits and percent reduction in total dry weight. Figure S4: Individual PCA plot for salt tolerance index-related traits and percent reduction in total dry weight in 27 tomato genotypes at the seedling stage under salinized (200 mM NaCl) and non-salinized conditions during 2018–2019.
