# Supplementary material for: Early Growth Stage Characterization and the Biochemical Responses for Salinity Stress in Tomato
Source: Plants (Basel). 2021 Apr 7;10(4):712. doi: 10.3390/plants10040712 (PMC8067703; doi:10.3390/plants10040712)
Supplement: Supplementary file 1 [file plants-10-00712-s001.pdf]

**Table S1. Analysis of variance (ANOVA) (mean square) for physiomorphological traits in 27 tomato genotypes at the seedling stage under non-salinized control (S1) with 18.2 mM NaCl and salinized (S2) with 200mM NaCl conditions during 2018-2019**

| SOV | df | TLA | NLF | SPAD1 | SPAD4 | Chla | Chlb | LRWC | MSI | SHTLN | RTLN | RSLR | SDW | RDW | RTSDW | Na | K | Ca | KTNa |
|---|---|---|---|---|---|---|---|---|---|---|---|---|---|---|---|---|---|---|---|
| **Replication** | 3 | 6115.94 | 7.25 | 138.78 | 34.55 | 0.28 | 0.025 | 40.56 | 12.86 | 92.01 | 3.26 | 0.002 | 4.78 | 0.001 | 0.0002 | 0.009 | 0.004 | 0.0290 | 0.09 |
| **Treatment (A)** | 1 | 4673605.30 | 4854.51 | 125.88 | 4372.20 | 3.77 | 1.426 | 8149.99 | 6584.69 | 43449.22 | 2827.51 | 0.048 | 9953.73 | 22.53 | 0.022 | 189.07 | 133.02 | 41.1393 | 1350.2 |
| **Genotypes (B)** | 26 | 49709.03 | 34.61 | 72.64 | 81.91 | 0.25 | 0.055 | 159.56 | 13.25 | 326.75 | 54.01 | 0.007 | 19.71 | 0.20 | 0.001 | 1.82 | 0.356 | 0.1749 | 6.69 |
| **AxB** | 26 | 10418.66 | 18.42 | 9.11 | 27.67 | 0.07 | 0.028 | 31.73 | 8.57 | 105.30 | 47.29 | 0.011 | 7.99 | 0.135 | 0.0007 | 1.52 | 0.34 | 0.0857 | 5.86 |
| **Error** | 159 | 1065.58 | 4.74 | 8.45 | 17.59 | 0.001 | 0.005 | 0.35 | 0.041 | 41.05 | 9.72 | 0.002 | 2.15 | 0.016 | 0.0002 | 0.011 | 0.004 | 0.0686 | 0.35 |
| **CV** | | 6.34 | 13.89 | 6.41 | 10.34 | 4.53 | 11.97 | 0.82 | 0.50 | 8.88 | 13.23 | 16.33 | 11.61 | 16.64 | 20.99 | 7.71 | 5.13 | 16.17 | 21.37 |

** and *** indicates significant at 1% and 0.1% level of probability respectively

**TLA=** Total Leaf Area per plant ($cm^2$), **NLF=** Number of leaves per plant**, SPAD1=** SPAD value at first week of salt stress**, SPAD4=** SPAD value at fourth week of salt stress**, Chla=** Chlorophyll a concentration of fresh leaves(mg $g^{-1}$ fresh mass)**, Chlb=** Chlorophyll b concentration of fresh leaves (mg $g^{-1}$ fresh mass)**, LRWC =** Leaf relative water content(%)**, MSI=** Membrane stability index of fresh leaves, **SHTLN=** Shoot length(cm)**, RTLN=** Root length(cm), **RSLR=** Root to shoot length ratio **, SDW=**Shoot dry weight per plant(g), **RDW=**Root dry weight per plant (g), **RSDW** = Root to shoot dry weight ratio, **Na**= Sodium content in leaf (mmol/g DW), **K**= Potassium content in leaf (mmol/g DW), **Ca**= Calcium content in leaf (mmol/g DW), **KTNa**= Potassium to sodium content ratio in leaf

**Supplementary Figure S1.** Effect of salinity on 18 physio-morphological traits in 27 tomato genotypes at the seedling stage under non-salinized control (S1) with 18.2 mM NaCl and salinized (S2) with 200mM NaCl conditions during 2018-2019. Each bar represents four replicates mean value along with standard error.

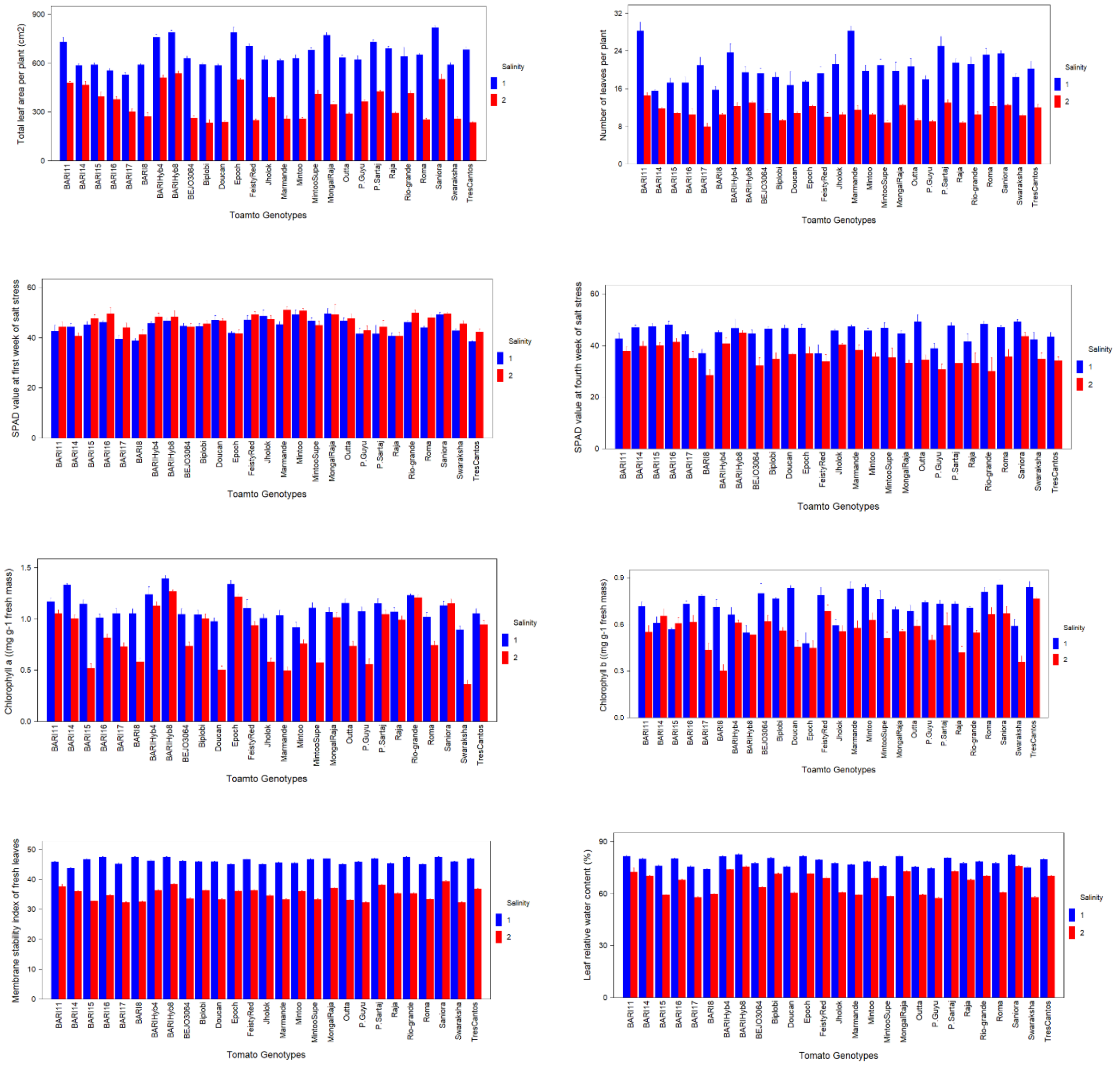

**Supplementary Figure S1 (continued).** Effect of salinity on 18 physio-morphological traits in 27 tomato genotypes at the seedling stage under non-salinized control (S1) with 18.2 mM NaCl and salinized (S2) with 200mM NaCl conditions during 2018-2019. Each bar represents four replicates mean value along with standard error

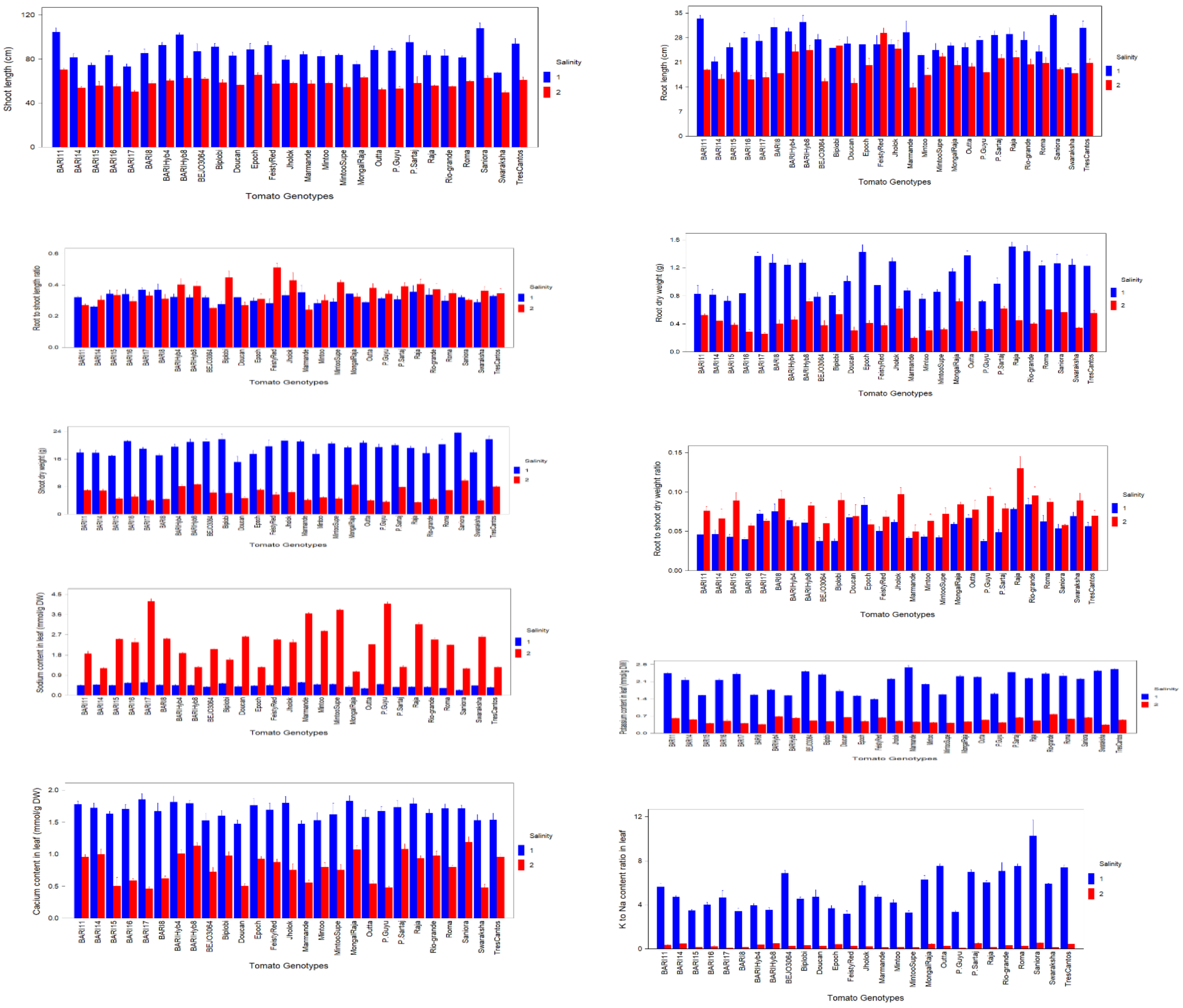

**Supplementary Figure S2.** Visual salt damage score for severity of salt susceptibility by on a 1–5 scale (Dasgan et al. 2002) and percent reduction in total dry weight of 18 physio-morphological traits in 27 tomato genotypes at the seedling stage under 200mM NaCl conditions during 2018-2019. Each bar represents four replicates mean value along with standard error.

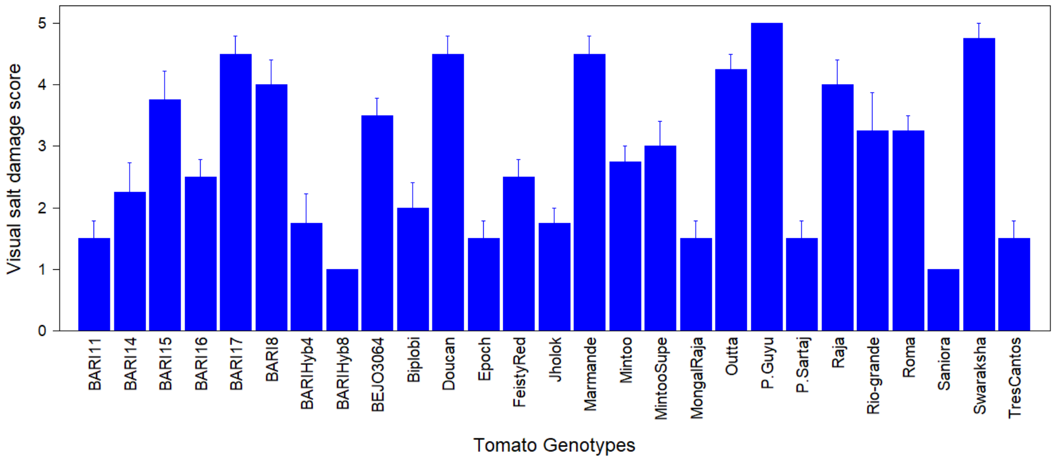


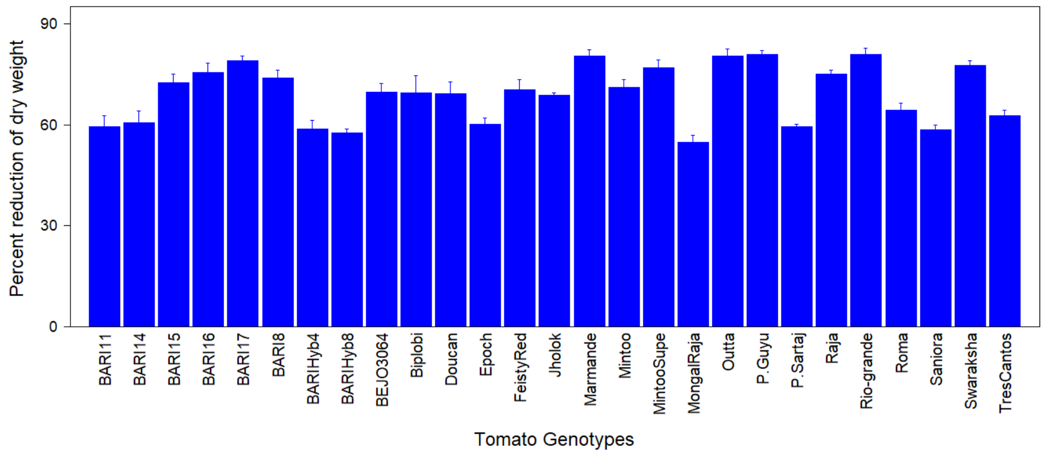

**Table S2. Correlation co-efficient between 18 physio-morphological traits in 27 tomato genotypes at the seedling stage under non-salinized control (S1) with 18.2 mM NaCl and salinized (S2) with 200mM NaCl conditions during 2018-2019**

| | NLF | SPAD1 | SPAD4 | Chla | Chlb | LRWC | MSI | SHTLN | RTLN | RSR | SDW | RDW | RTSDW | Na | K | Ca | KTNa |
|---|---|---|---|---|---|---|---|---|---|---|---|---|---|---|---|---|---|
| **TLA** | 0.86** | -0.12 | 0.75** | 0.73** | 0.62** | 0.88** | 0.91** | 0.88** | 0.76** | -0.21 | 0.89** | 0.8** | -0.51** | -0.81** | 0.81** | 0.91** | 0.81** |
| **NLF** | | -0.16 | 0.76** | 0.57** | 0.67** | 0.79** | 0.88** | 0.88** | 0.72** | -0.29 | 0.9** | 0.79** | -0.53** | -0.81** | 0.9** | 0.86** | 0.87** |
| **SPAD1** | | | 0.05 | -0.14 | 0.01 | -0.1 | -0.19 | -0.19 | -0.2 | 0.01 | -0.19 | -0.26 | -0.1 | 0.19 | -0.24 | -0.22 | -0.19 |
| **SPAD4** | | | | 0.54** | 0.56** | 0.73** | 0.79** | 0.72** | 0.55** | -0.29* | 0.81** | 0.69** | -0.55** | -0.73** | 0.78** | 0.75** | 0.77** |
| **Chla** | | | | | 0.37** | 0.87** | 0.69** | 0.64** | 0.61** | -0.06 | 0.62** | 0.61** | -0.3* | -0.73** | 0.52** | 0.76** | 0.5** |
| **Chlb** | | | | | | 0.6** | 0.68** | 0.63** | 0.56** | -0.14 | 0.69** | 0.51** | -0.58** | -0.65** | 0.67** | 0.62** | 0.65** |
| **LRWC** | | | | | | | 0.9** | 0.83** | 0.72** | -0.2 | 0.85** | 0.76** | -0.49** | -0.89** | 0.77** | 0.92** | 0.74** |
| **MSI** | | | | | | | | 0.9** | 0.77** | -0.24 | 0.97** | 0.86** | -0.54** | -0.9** | 0.91** | 0.97** | 0.87** |
| **SHTLN** | | | | | | | | | 0.81** | -0.33* | 0.92** | 0.78** | -0.55** | -0.81** | 0.83** | 0.88** | 0.84** |
| **RTLN** | | | | | | | | | | 0.29* | 0.76** | 0.72** | -0.27 | -0.67** | 0.69** | 0.78** | 0.7** |
| **RSR** | | | | | | | | | | | -0.29* | -0.14 | 0.46** | 0.25 | -0.25 | -0.19 | -0.27 |
| **SDW** | | | | | | | | | | | | 0.86** | -0.59** | -0.88** | 0.93** | 0.95** | 0.91** |
| **RDW** | | | | | | | | | | | | | -0.18 | -0.81** | 0.82** | 0.88** | 0.84** |
| **RTSDW** | | | | | | | | | | | | | | 0.49** | -0.52** | -0.47** | -0.45** |
| **Na** | | | | | | | | | | | | | | | -0.8** | -0.9** | -0.78** |
| **K** | | | | | | | | | | | | | | | | 0.88** | 0.94** |
| **Ca** | | | | | | | | | | | | | | | | | 0.84** |

**df = 54-2 = 52; r0.05 = 0.273, r0.01 = 0.354, ** Significant at 1% level, * Significant at 5% level.**

**Where, TLA=** Total Leaf Area per plant ($cm^2$), **NLF=** Number of leaves per plant**, SPAD1=** SPAD value at first week of salt stress**, SPAD4=** SPAD value at fourth week of salt stress**, Chla=** Chlorophyll a concentration of fresh leaves(mg $g^{-1}$ fresh mass)**, Chlb=** Chlorophyll b concentration of fresh leaves (mg $g^{-1}$ fresh mass )**, LRWC =** Leaf relative water content(%)**, MSI=** Membrane stability index of fresh leaves, **SHTLN=** Shoot length(cm)**, RTLN=** Root length(cm), **RSLR=** Root to shoot length ratio**, SDW=**Shoot dry weight per plant (g), **RDW=**Root dry weight per plant (g.), **RSDW** = Root to shoot dry weight ratio, **Na**= Sodium content in leaf (mmol/g DW), **K**= Potassium content in leaf (mmol/g DW), **Ca**= Calcium content in leaf (mmol/g DW), **KTNa**= Potassium to sodium content ratio in leaf

**Table S3. Principal component (PC) loadings of physio-morphological traits among in 27 tomato genotypes at the seedling stage under non-salinized control (S1) with 18.2 mM NaCl and salinized (S2) with 200mM NaCl conditions during 2018-2019**

| Traits | PC1 | PC2 | PC3 | PC4 | PC5 | PC6 | PC7 | PC8 | PC9 | PC10 |
|---|---|---|---|---|---|---|---|---|---|---|
| **Std deviation** | 3.556 | 1.264 | 0.865 | 0.792 | 0.622 | 0.576 | 0.439 | 0.391 | 0.318 | 0.270 |
| **Proportion of variation** | 0.744 | 0.094 | 0.044 | 0.037 | 0.023 | 0.020 | 0.011 | 0.009 | 0.006 | 0.004 |
| **Cumulative proportion** | 0.744 | 0.838 | 0.882 | 0.919 | 0.941 | 0.961 | 0.972 | 0.981 | 0.987 | 0.992 |
| **TLA** | -0.256 | -0.049 | 0.002 | -0.138 | 0.084 | -0.086 | 0.654 | 0.608 | 0.261 | -0.122 |
| **NLF** | -0.265 | 0.085 | -0.052 | 0.173 | -0.015 | 0.098 | 0.195 | 0.150 | -0.655 | 0.555 |
| **SPAD4** | -0.229 | 0.146 | 0.048 | 0.174 | 0.274 | -0.847 | 0.088 | -0.280 | -0.025 | -0.016 |
| **Chla** | -0.210 | -0.199 | -0.123 | -0.729 | -0.083 | -0.114 | -0.069 | -0.166 | -0.259 | -0.251 |
| **Chlb** | -0.200 | 0.084 | 0.585 | 0.025 | -0.756 | -0.082 | 0.116 | -0.122 | 0.046 | -0.017 |
| **LRWC** | -0.260 | -0.067 | -0.087 | -0.380 | -0.008 | -0.061 | -0.056 | -0.046 | -0.024 | 0.338 |
| **MSI** | -0.276 | -0.010 | -0.027 | 0.039 | 0.047 | 0.062 | -0.128 | 0.112 | 0.188 | -0.121 |
| **SHTLN** | -0.264 | 0.061 | -0.052 | 0.074 | 0.139 | 0.342 | 0.345 | -0.469 | 0.012 | -0.160 |
| **RTLN** | -0.222 | -0.400 | 0.225 | 0.131 | 0.255 | 0.255 | 0.219 | -0.344 | -0.019 | -0.085 |
| **RSLR** | 0.061 | -0.700 | 0.421 | 0.087 | 0.181 | -0.127 | -0.186 | 0.184 | -0.050 | 0.106 |
| **SDW** | -0.277 | 0.040 | -0.013 | 0.095 | 0.029 | 0.046 | -0.185 | -0.013 | 0.239 | 0.174 |
| **RDW** | -0.253 | -0.192 | -0.293 | 0.225 | -0.183 | -0.034 | -0.110 | -0.065 | 0.240 | 0.180 |
| **RSDW** | 0.160 | -0.459 | -0.532 | 0.222 | -0.425 | -0.174 | 0.235 | -0.078 | -0.097 | -0.112 |
| **Na** | 0.273 | -0.018 | 0.130 | -0.112 | 0.050 | -0.034 | 0.186 | -0.004 | -0.226 | 0.009 |
| **K** | -0.267 | 0.044 | 0.007 | 0.215 | -0.005 | 0.031 | -0.238 | 0.261 | -0.434 | -0.517 |
| **Ca** | -0.270 | -0.109 | -0.090 | -0.138 | -0.040 | 0.063 | -0.221 | 0.088 | 0.157 | 0.196 |
| **KTNa** | -0.275 | 0.029 | -0.069 | 0.162 | -0.033 | 0.031 | -0.212 | 0.121 | -0.075 | -0.242 |

**TLA=** Total Leaf Area per plant ($cm^2$), **NLF=** Number of leaves per plant**, SPAD1=** SPAD value at first week of salt stress**, SPAD4=** SPAD value at fourth week of salt stress**, Chla=** Chlorophyll a concentration of fresh leaves(mg $g^{-1}$ fresh mass)**, Chlb=** Chlorophyll b concentration of fresh leaves (mg $g^{-1}$ fresh mass)**, LRWC =** Leaf relative water content(%)**, MSI=** Membrane stability index of fresh leaves, **SHTLN=** Shoot length(cm)**, RTLN=** Root length(cm), **RSLR=** Root to shoot length ratio **, SDW=**Shoot dry weight per plant(g), **RDW=**Root dry weight per plant (g), **RSDW** = Root to shoot dry weight ratio, **Na**= Sodium content in leaf (mmol/g DW), **K**= Potassium content in leaf (mmol/g DW), **Ca**= Calcium content in leaf (mmol/g DW), **KTNa**= Potassium to sodium content ratio in leaf

**Table S4. Analysis of variance (ANOVA) (mean square) for salt tolerance indices related traits in 27 tomato genotypes at the seedling stage under non-salinized control (S1) with 18.2 mM NaCl and salinized (S2) with 200mM NaCl conditions during 2018-2019**

| SOV | df | Score | TOL | SSI | PREDFRW | PREDDM | MPI | HMI | GMPI | SDWSTI | RDWSTI | TDMSTI | K Na STI | SDWSI | RDWSI | TDMSI | BMYI |
|---|---|---|---|---|---|---|---|---|---|---|---|---|---|---|---|---|---|
| **Replication** | 3 | 2.0586 | 9.0343 | 0.0105 | 49.3704 | 50.4468 | 2.3407 | 2.4826 | 2.0368 | 0.0051 | 0.0022 | 0.0049 | 0.0001 | 0.0048 | 0.0334 | 0.0050 | 0.0421 |
| **Genotypes** | 26 | 6.5064 | 16.9974 | 0.0589 | 265.5449 | 282.8682 | 10.6768 | 21.9869 | 16.4680 | 0.0507 | 0.1272 | 0.0513 | 0.0070 | 0.0282 | 0.0818 | 0.0283 | 0.3735 |
| **Error** | 78 | 0.3792 | 3.6020 | 0.0043 | 45.3626 | 20.6336 | 1.2793 | 1.0953 | 1.0586 | 0.0031 | 0.0100 | 0.0030 | 0.0001 | 0.0022 | 0.0114 | 0.0021 | 0.0168 |
| **CV** | | 21.52 | 13.34 | 6.56 | 11.17 | 6.56 | 8.43 | 11.03 | 9.15 | 18.18 | 24.40 | 17.44 | 20.29 | 15.49 | 25.10 | 14.76 | 12.98 |

** and *** indicate significant at 1% and 0.1% level of probability respectively

**Here, Score =** Visual salt damage score, **TOL =** Tolerance Index**, SSI =** Stress susceptibility index**, PREDFRW =** Percent reduction of fresh weight**, PREDDM =** Percent reduction of dry weight**, MPI =** Mean productivity index**, HMI =** Harmonic mean index**, GMPI =** Geometric mean productivity index, **SDWSTI =** Shoot dry weight stress tolerance index**, RDWSTI =** Root dry weight stress tolerance index, **TDMSTI =** Total dry matter stress tolerance index **, KNaSTI =**Potassium to sodium ion ration tolerance index, **SDWSI=**Shoot dry weight stress index, **RDWSI** = Root dry weight stress index, **TDMSI** = Total dry matter stress index, **BMYI** = Biomass yield index

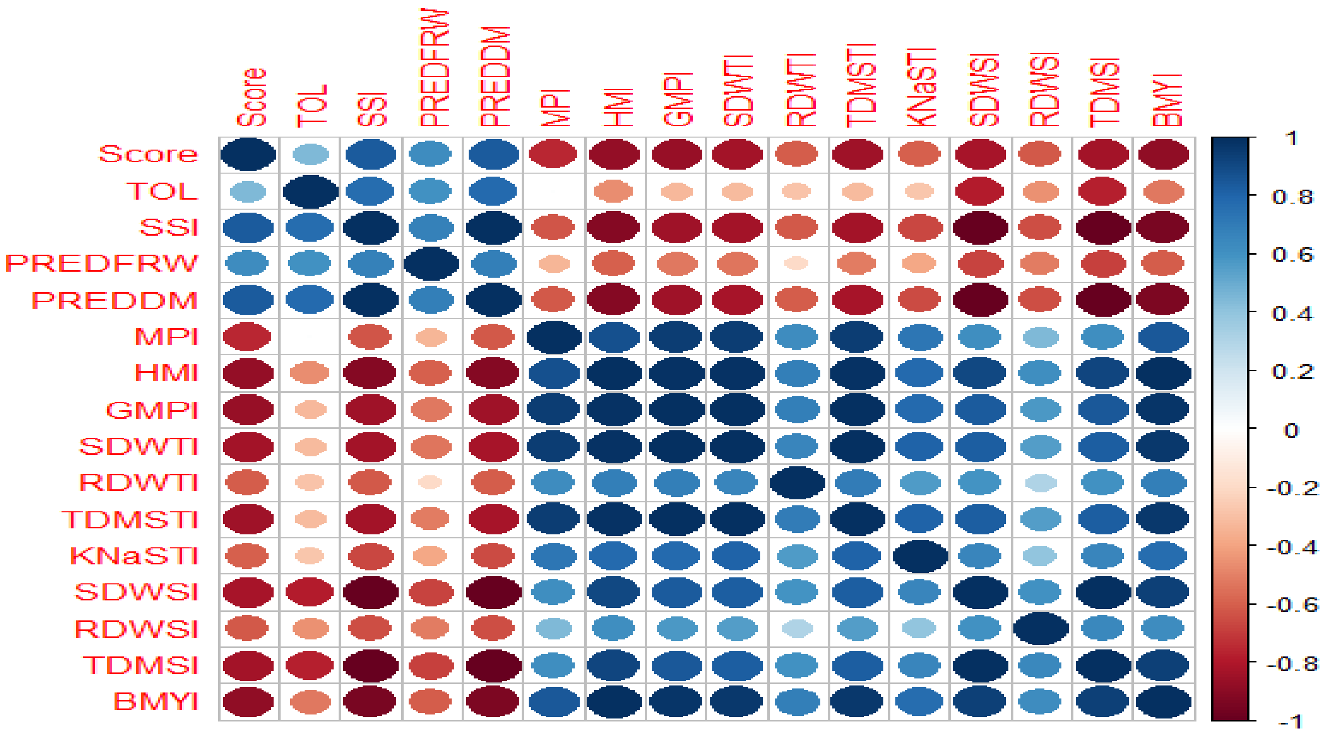


Supplementary **Figure S3. Correlation co-efficient between salt tolerance indices related traits in 27 tomato genotypes at the seedling stage under non-salinized control (S1) with 18.2 mM NaCl and salinized (S2) with 200mM NaCl conditions during 2018-2019. Where, Score = Visual salt damage score, TOL = Stress tolerance, SSI = Stress susceptibility index, PREDFRW = Percent reduction of fresh weight, PREDDM = Percent reduction of dry weight, MPI = Mean productivity index, HMI = Harmonic mean index, GMPI = Geometric mean productivity index, SDWSTI = Shoot dry weight stress tolerance index, RDWSTI = Root dry weight stress tolerance index, TDMSTI = Total dry matter stress tolerance index , KNaSTI =Potassium to sodium ion ration tolerance index, SDWSI=Shoot dry weight stress index, RDWSI = Root dry weight stress index, TDMSI = Total dry matter stress intensity, BMYI = Biomass yield index. Pearson correlation matrix of the studied tolerance indices attributes under salinity (200mM NaCl) relative to control conditions (n =4). Large circles represent strong correlations and smaller circles represent weaker correlations. The color scale indicates the extent of correlation, where 1 denotes completely positive correlation (dark blue) and -1 denotes completely negative correlation (dark red) between two traits. Only significant correlations are shown (P=0.05).**

**Table S5. Principal component (PC) loadings for salt tolerance indices related traits in 27 tomato genotypes at the seedling stage under non-salinized control (S1) with 18.2 mM NaCl and salinized (S2) with 200mM NaCl conditions during 2018-2019**

| Traits | PC1 | PC2 | PC3 | PC4 | PC5 | PC6 | PC7 | PC8 | PC9 | PC10 |
|---|---|---|---|---|---|---|---|---|---|---|
| **Std deviation** | 3.480 | 1.326 | 0.922 | 0.685 | 0.590 | 0.558 | 0.354 | 0.161 | 0.041 | 0.017 |
| **Proportion of variation** | 0.757 | 0.110 | 0.053 | 0.029 | 0.022 | 0.019 | 0.008 | 0.002 | 0.000 | 0.000 |
| **Cumulative proportion** | 0.757 | 0.867 | 0.920 | 0.949 | 0.971 | 0.990 | 0.998 | 1.000 | 1.000 | 1.000 |
| **Score** | -0.263 | -0.077 | -0.070 | -0.027 | 0.134 | 0.398 | -0.838 | 0.194 | -0.046 | 0.013 |
| **TOL** | -0.167 | 0.586 | 0.241 | 0.011 | 0.127 | -0.103 | -0.058 | -0.163 | -0.577 | -0.182 |
| **SSI** | -0.277 | 0.160 | 0.103 | -0.015 | 0.139 | -0.091 | -0.078 | -0.440 | 0.289 | 0.347 |
| **PREDFRW** | -0.191 | 0.315 | -0.418 | -0.399 | -0.467 | 0.501 | 0.212 | -0.126 | 0.019 | -0.010 |
| **PREDDM** | -0.276 | 0.171 | 0.100 | -0.013 | 0.144 | -0.093 | -0.069 | -0.451 | 0.222 | -0.083 |
| **MPI** | 0.225 | 0.452 | 0.147 | 0.044 | -0.066 | 0.020 | -0.025 | 0.392 | -0.176 | 0.377 |
| **HMI** | 0.284 | 0.088 | 0.048 | 0.036 | -0.089 | 0.050 | -0.146 | -0.205 | 0.215 | 0.290 |
| **GMPI** | 0.274 | 0.215 | 0.082 | 0.040 | -0.085 | 0.041 | -0.111 | -0.004 | 0.144 | 0.276 |
| **SDWTI** | 0.272 | 0.205 | 0.138 | 0.088 | -0.094 | 0.096 | -0.123 | -0.031 | 0.286 | -0.560 |
| **RDWTI** | 0.190 | 0.174 | -0.636 | -0.321 | 0.144 | -0.576 | -0.259 | 0.021 | 0.008 | -0.045 |
| **TDMSTI** | 0.274 | 0.211 | 0.084 | 0.051 | -0.073 | 0.042 | -0.132 | -0.019 | 0.258 | -0.385 |
| **KNaSTI** | 0.227 | 0.122 | -0.295 | 0.121 | 0.754 | 0.441 | 0.256 | -0.051 | 0.014 | 0.007 |
| **SDWSI** | 0.273 | -0.205 | -0.077 | 0.089 | -0.124 | 0.088 | -0.120 | -0.404 | -0.387 | -0.004 |
| **RDWSI** | 0.195 | -0.147 | 0.430 | -0.835 | 0.225 | 0.065 | 0.011 | 0.003 | -0.016 | -0.012 |
| **TDMSI** | 0.275 | -0.204 | -0.052 | 0.014 | -0.099 | 0.077 | -0.109 | -0.370 | -0.354 | -0.027 |
| **BMYI** | 0.286 | 0.033 | 0.026 | 0.034 | -0.095 | 0.056 | -0.113 | -0.150 | 0.101 | 0.270 |

**Where, Score =** Visual salt damage score, **TOL =** Stress tolerance**, SSI =** Stress susceptibility index**, PREDFRW =** Percent reduction of fresh weight**, PREDDM =** Percent reduction of dry weight**, MPI =** Mean productivity index**, HMI =** Harmonic mean index**, GMPI =** Geometric mean productivity index, **SDWSTI =** Shoot dry weight stress tolerance index**, RDWSTI =** Root dry weight stress tolerance index, **TDMSTI =** Total dry matter stress tolerance index **, KNaSTI =**Potassium to sodium ion ration tolerance index, **SDWSI=**Shoot dry weight stress index, **RDWSI** = Root dry weight stress index, **TDMSI** = Total dry matter stress intensity, **BMYI** = Biomass yield index

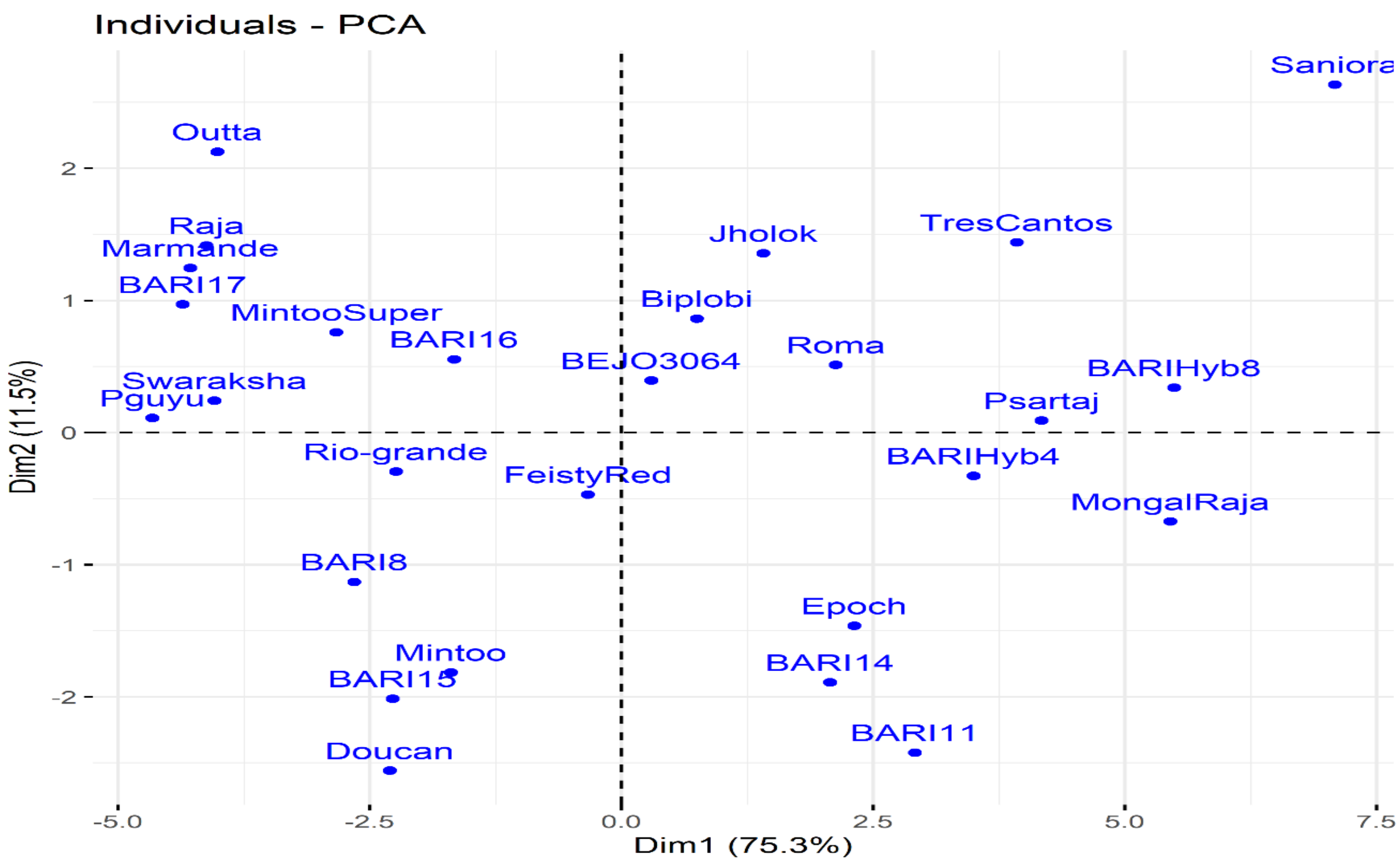


**Supplementary Fig S4 Individuals PCA plot for salt tolerance indices related traits in 27 tomato genotypes at the seedling stage under non-salinized control (S1) with 18.2 mM NaCl and salinized (S2) with 200mM NaCl conditions during 2018-2019.**

**Table S6. Individual coordinates-PC scores for salt tolerance indices related traits in 27 tomato genotypes at the seedling stage under non-salinized control (S1) with 18.2 mM NaCl and salinized (S2) with 200mM NaCl conditions during 2018-2019**

| Genotypes | Dim.1 | Dim.2 | Dim.3 | Dim.4 | Dim.5 | Ranking genotypes for salt tolerance |
|---|---|---|---|---|---|---|
| BARI8 | -2.65603 | -1.13057 | 0.583232 | -0.12235 | -0.4618 | 20 |
| BARI11 | 2.914482 | -2.42157 | -0.94988 | 0.274178 | 0.071703 | 7 |
| BARI14 | 2.068687 | -1.89104 | -0.1596 | -0.02278 | 0.723592 | 10 |
| BARI15 | -2.276 | -2.01439 | -0.74672 | 0.705128 | 0.414541 | 18 |
| BARI16 | -1.66218 | 0.554677 | -1.56761 | -0.90151 | -0.79942 | 15 |
| BARI17 | -4.35891 | 0.969832 | 0.969342 | -0.07562 | 0.064116 | 26 |
| BARIHyb4 | 3.499709 | -0.32758 | 0.791918 | -0.44057 | -0.66152 | 6 |
| BARIHyb8 | 5.490627 | 0.339592 | 0.316713 | 0.645629 | -1.18183 | 2 |
| Epoch | 2.309893 | -1.46196 | 1.183964 | -0.85919 | -0.91652 | 8 |
| Biplobi | 0.748964 | 0.861659 | -1.40877 | 1.413949 | 0.12546 | 12 |
| MongalRaja | 5.449915 | -0.67284 | 0.735926 | 0.799578 | 0.284098 | 3 |
| Mintoo | -1.69375 | -1.81525 | -0.66997 | -0.43364 | -0.32137 | 16 |
| MintooSuper | -2.83282 | 0.759478 | -0.65879 | 0.310865 | -0.37705 | 21 |
| Jholok | 1.405152 | 1.357689 | 0.178695 | 0.939574 | -1.10785 | 11 |
| Swaraksha | -4.04517 | 0.242377 | 1.190802 | 0.390269 | 0.513497 | 23 |
| Rio-grande | -2.24224 | -0.29393 | 0.754974 | -0.82353 | 0.231583 | 17 |
| Raja | -4.12595 | 1.416752 | 1.056769 | 0.970282 | -0.3922 | 24 |
| P.Sartaj | 4.171478 | 0.090538 | 0.028986 | 0.775378 | 1.263867 | 4 |
| P.Guyu | -4.66299 | 0.112036 | -0.91847 | 0.542907 | 0.466979 | 27 |
| Roma | 2.127493 | 0.513264 | 0.65581 | 0.535279 | 0.099394 | 9 |
| TresCantos | 3.925255 | 1.439323 | 0.122384 | -0.28962 | 0.148807 | 5 |
| Marmande | -4.28464 | 1.245559 | -0.60445 | -0.85173 | -0.04375 | 25 |
| FeistyRed | -0.33389 | -0.46927 | -0.93423 | -0.67821 | -0.87767 | 14 |
| BEJO3064 | 0.297227 | 0.394294 | -1.31937 | -0.29007 | 0.473129 | 13 |
| Outta | -4.01631 | 2.125059 | 0.620984 | -0.24853 | 0.591441 | 22 |
| Doucan | -2.30122 | -2.55831 | 1.023389 | -0.68004 | 0.723863 | 19 |
| Saniora | 7.083223 | 2.634586 | -0.27602 | -1.58563 | 0.944909 | 1 |
